# Supplementary figures and images for: Genetic Iron Overload Hampers Development of Cutaneous Leishmaniasis in Mice
Source: Int J Mol Sci. 2023 Jan 14;24(2):1669. doi: 10.3390/ijms24021669 (PMC9864902; doi:10.3390/ijms24021669)

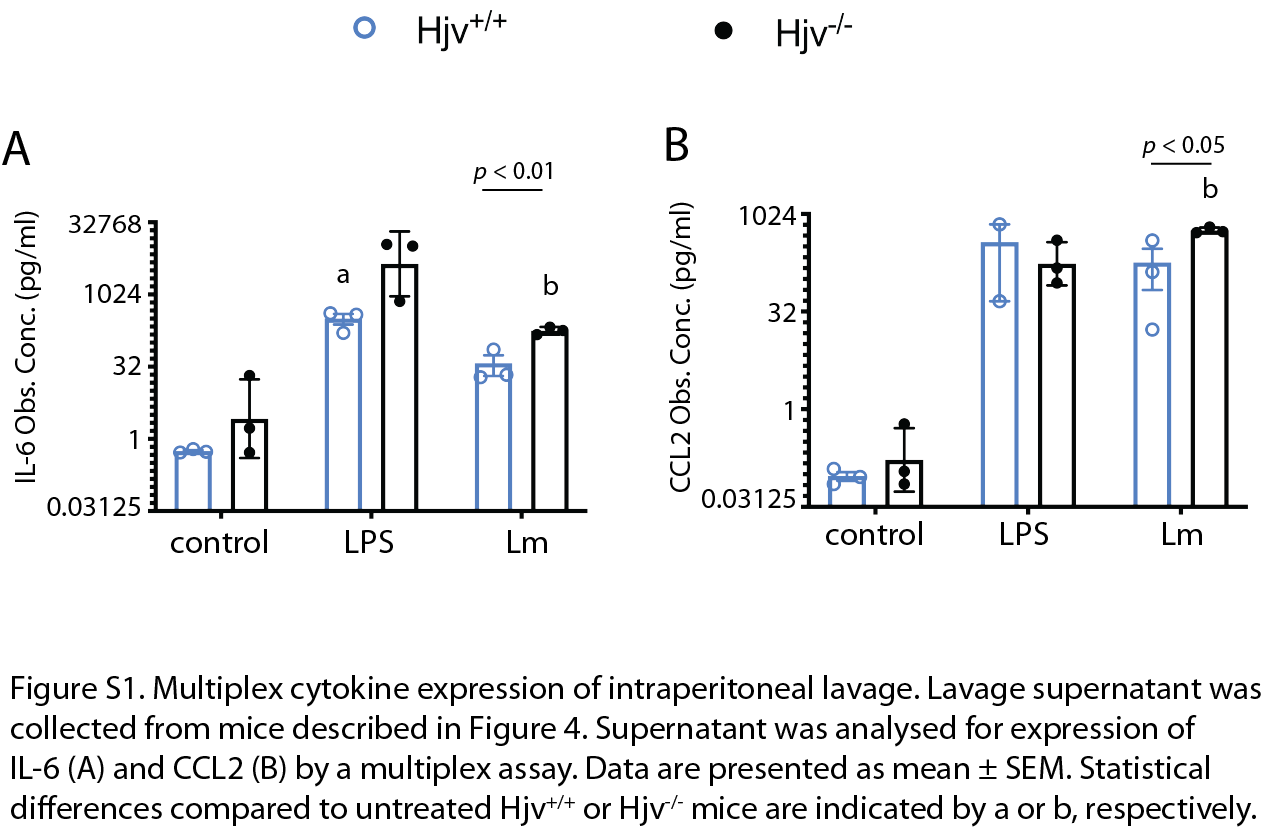

Supplement: Supplementary file 1 [file ijms-24-01669-s001.zip › ijms-2112431-supplementary.tif]
